# Energy restriction induced SIRT6 inhibits microglia activation and promotes angiogenesis in cerebral ischemia via transcriptional inhibition of TXNIP

**DOI:** 10.1038/s41419-022-04866-x

**Published:** 2022-05-11

**Authors:** Ming-Yu Song, Fang Yi, Hui Xiao, Jun Yin, Qing Huang, Jian Xia, Xiao-Meng Yin, Yan-Bin Wen, Le Zhang, Yun-Hai Liu, Bo Xiao, Wen-Ping Gu

**Affiliations:** 1 Department of Neurology, Xiangya Hospital, Central South University, Changsha, 410008 Hunan Province P.R. China; 2 National Clinical Research Center for Geriatric Disorders, Xiangya Hospital, Central South University, Changsha, 410008 Hunan Province P.R. China; 3 Clinical Research Center for Cerebrovascular Disease of Hunan Province, Central South University, Changsha, 410008 Hunan Province P.R. China; 4 Department of Geriatric Neurology, Xiangya Hospital, Central South University, Changsha, 410008 Hunan Province P.R. China; 5 Department of Neurology, Changsha Central Hospital, Changsha, 410004 Hunan Province P.R. China; 6 Department of Integrated Traditional Chinese and Western Medicine, Xiangya Hospital, Central South University, Changsha, 410008 Hunan Province P.R. China

**Keywords:** Diseases, Cardiovascular diseases

## Abstract

Energy restriction (ER) protects against cerebral ischemic injury, but the underlying mechanism remains largely unclear. Here, rats were fed *ad libitum* (AL) or on an alternate-day food deprivation intermittent fasting (IF) diet for 3 months, followed by middle cerebral artery occlusion (MCAO) surgery. The body weight, infarct volume, and neurological deficit score were accessed at the designated time points. ELISA, qRT-PCR, and Western blotting were used to determine cytokine secretion and the expression of SIRT6, TXNIP, and signaling molecules, respectively. Immunofluorescence evaluated microglial activation and angiogenesis in vivo. For in vitro study, oxygen-glucose deprivation/reoxygenation (OGD/R)-treated cell model was generated. MTT and tube formation assays were employed to determine cell viability and tube formation capability. ChIP assay detected chromatin occupancy of SIRT6 and SIRT6-mediated H3 deacetylation. We found that IF or ER mimetics ameliorated cerebral ischemic brain damage and microglial activation, and potentiated angiogenesis in vivo. ER mimetics or SIRT6 overexpression alleviated cerebral ischemia and reperfusion (I/R)-induced injury in vitro. SIRT6 suppressed TXNIP via deacetylation of H3K9ac and H3K56ac in HAPI cells and BMVECs. Downregulation of SIRT6 reversed ER mimetics-mediated protection during cerebral I/R in vitro. Our study demonstrated that ER-mediated upregulation of SIRT6 inhibited microglia activation and potentiated angiogenesis in cerebral ischemia via suppressing TXNIP.

## Introduction

Energy restriction (ER) has been shown to extend lifespan in various organisms ranging from yeast to rodents and primates [1]. ER is also involved in preventing age-related diseases and improving acute stress responses [2]. Intermittent fasting (IF) improves the outcome of ischemic brain injury by regulating neurotrophic factors, antioxidant enzymes, and inflammatory pathways in young mice [3]. A study has illustrated that IF protected against brain damage after cerebral ischemia and reperfusion (I/R) by modulating neurogenesis [4]. However, the detailed underlying mechanism by which IF protects against neurological damage after ischemic stroke remains elusive.

Sirtuin6 (SIRT6), a member of sirtuin family of NAD^+^-dependent histone deacetylases, plays important roles in diverse biological processes [5]. SIRT6 is ubiquitously expressed in most mouse tissues with high levels in thymus, skeletal muscle and brain [6], in particular, SIRT6 is widely expressed throughout the brain [7]. Cerebral I/R-induced SIRT6 reduction was associated with release of pro-inflammatory cytokine high mobility group box-1 (HMGB1) [7], as well as nuclear factor (erythroid-derived 2)-like 2 (NRF2) activation [8]. It has been reported that SIRT6 exerted neuroprotective effects by regulating inflammation, the levels of antioxidant genes and degradation of senescence factors [9]. More importantly, a recent study has illustrated that a novel SIRT6 activator MDL-811 exerted anti-inflammatory effects in primary mouse microglial [10], supporting the neuroprotective role of SIRT6 in microglial. ER increased SIRT6 expression [11], suggesting that ER might exert neuroprotective effect via inducing SIRT6 after stroke.

SIRT6 suppresses thioredoxin-interacting protein (TXNIP) expression transcriptionally in pancreatic beta cells [12]. TXNIP contributes to redox homeostasis, glucose metabolism, inflammation, and angiogenesis [13]. Emerging evidence illustrated the pro-inflammatory role of TXNIP in NOD-like receptor protein 3 (NLRP3) inflammasome activation in different types of cells [13]. Upon activation, NLRP3 recruits apoptosis-associated speck-like adapter protein containing a CARD (ASC), and triggers caspase-1 activation, as well as maturation of IL-1β and IL-18 [14]. More importantly, TXNIP was increasingly implicated as a key regulator of angiogenesis [15, 16]. Indeed, the promigratory effects of angiogenic growth factors, such as VEGF, were mediated by their repression of TXNIP [16]. Therefore, we hypothesized that SIRT6/TXNIP axis might play important roles in ER-mediated neuroprotection after cerebral I/R.

In this study, we found that IF or ER mimetics protected against cerebral ischemia-induced impairment and microglial activation and potentiated middle cerebral artery occlusion (MCAO)-induced angiogenesis, and accompanied by changes in expression of SIRT6, TXNIP, and NLRP3 inflammasome. The in vitro studies suggested that ER mimetics or SIRT6 overexpression protected against oxygen-glucose deprivation/reoxygenation (OGD/R)-induced microglial activation in HAPI cells, whereas potentiated OGD/R-induced angiogenesis in BMVECs. Moreover, SIRT6 suppressed TXNIP via deacetylation of H3K9ac and H3K56ac in HAPI cells and BMVECs. These findings indicate that SIRT6 and its downstream molecule TXNIP may be the key players in ER-mediated neuroprotection.

## Results

### IF protects against cerebral ischemia-induced impairment and microglial activation whereas potentiated MCAO-induced angiogenesis

**Fig. 1:**
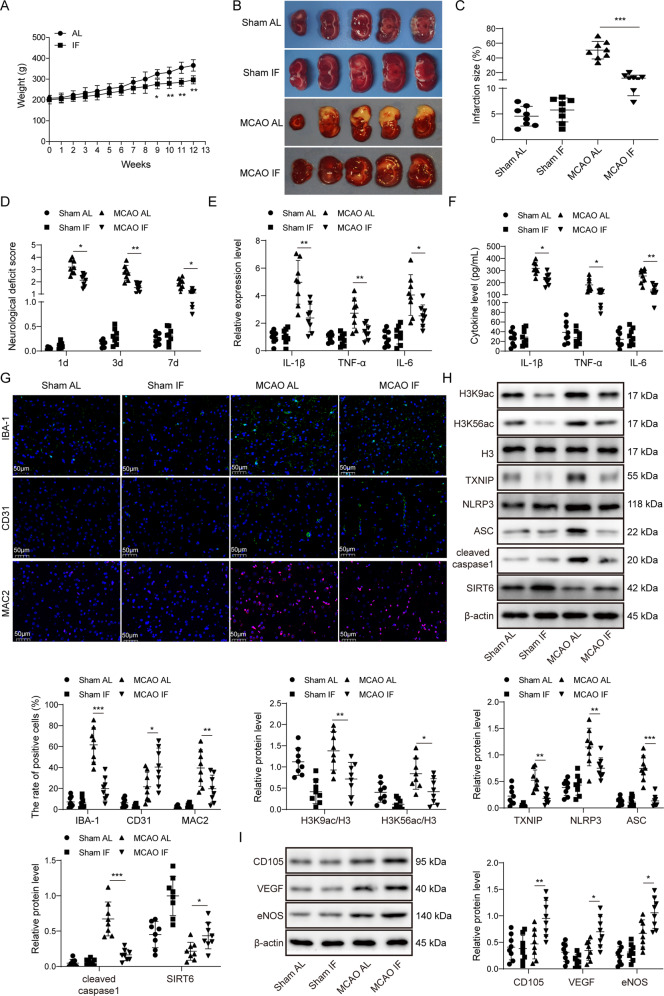
IF protects against cerebral ischemia-induced impairment, microglial activation, and potentiated MCAO-induced angiogenesis. **A** The body weights of rats. **B** TTC stained sections illustrating normal (red) and infarct area (white). **C** The size of infarct area was expressed as percentage (%) of brain volume. **D** Neurological deficit scores using Longa scoring system. **E** The mRNA levels of cytokines in brain tissues were determined by qRT-PCR. **F** The serum levels of cytokines were detected by ELISA assay. **G** Immunofluorescence staining of IBA-1 (Green), CD31 (Green) and MAC2 (Red) in the peri-infarct cortex. Blue, DAPI. Scale bar = 50 μm. **H**, **I** The protein levels of target proteins were determined by Western blotting. β-actin acted as a loading control for Western blotting. For animal studies, *n* = 8/group. *p < 0.05, **p < 0.01, and ***p < 0.001.

Rats were randomly divided into *ad libitum* (AL) and intermittent fasting (IF) groups. After 9 weeks, IF rats exhibited decreased body weight compared to AL rats (Fig. 1A). Both AL and IF rats were then assigned randomly to Sham and MCAO groups. The infarct area (white) was significantly increased in rats subjected to MCAO, and IF dramatically reduced the size of infarct area (Fig. 1B, C). The neurological deficit scores of MCAO rats were significantly higher than that of Sham rats, and the scores in MCAO IF group showed a remarkable reduction (Fig. 1D). The rescue effects of IF on infarcted area and neurological deficit scores were also accompanied by a decrease in the mRNA levels of IL-1β, TNF-α, and IL-6 in brain tissues compared to that of MCAO AL rats (Fig. 1E). Similar results were obtained for the serum levels of these cytokines (Fig. 1F). It was well-established that microglial activation is rapidly observed after MCAO, and it also reflected the severity of ischemic damage [17]. As expected, the immunofluorescence of the microglial marker ionized calcium binding adapter molecule (IBA-1) was more intense in MCAO AL and MCAO IF groups, while little IBA-1 was detected in Sham groups. IF significantly attenuated MCAO-induced IBA-1 expression (Fig. 1G). By contrast, IF further potentiated MCAO-mediated increase of the well-known vascular marker CD31 (Fig. 1G). The expression of MAC2, a marker of activated microglia, exhibited similar trend as IBA-1 (Fig. 1G). Increased levels of H3K9ac, H3K56ac, TXNIP, NLRP3, ASC and cleaved caspase-1 were found in MCAO AL and MCAO IF groups compared with control groups, whereas SIRT6 was significantly decreased in MCAO rats. More importantly, MCAO-induced changes of these proteins were partially abrogated by IF. No significant change of total H3 was observed (Fig. 1H). Furthermore, the vascularization markers CD105, the key angiogenesis contributor vascular endothelial growth factor (VEGF) and its downstream effector endothelial nitric oxide (eNOS) were also examined. As presented in Fig. 1I, MCAO-increased CD105, VEGF and eNOS were potentiated by IF. These findings suggest that IF ameliorates MCAO-induced impairment and microglial activation and potentiates MCAO-induced angiogenesis by increasing SIRT6.

### ER mimetics exert similar effects as IF in vivo

**Fig. 2:**
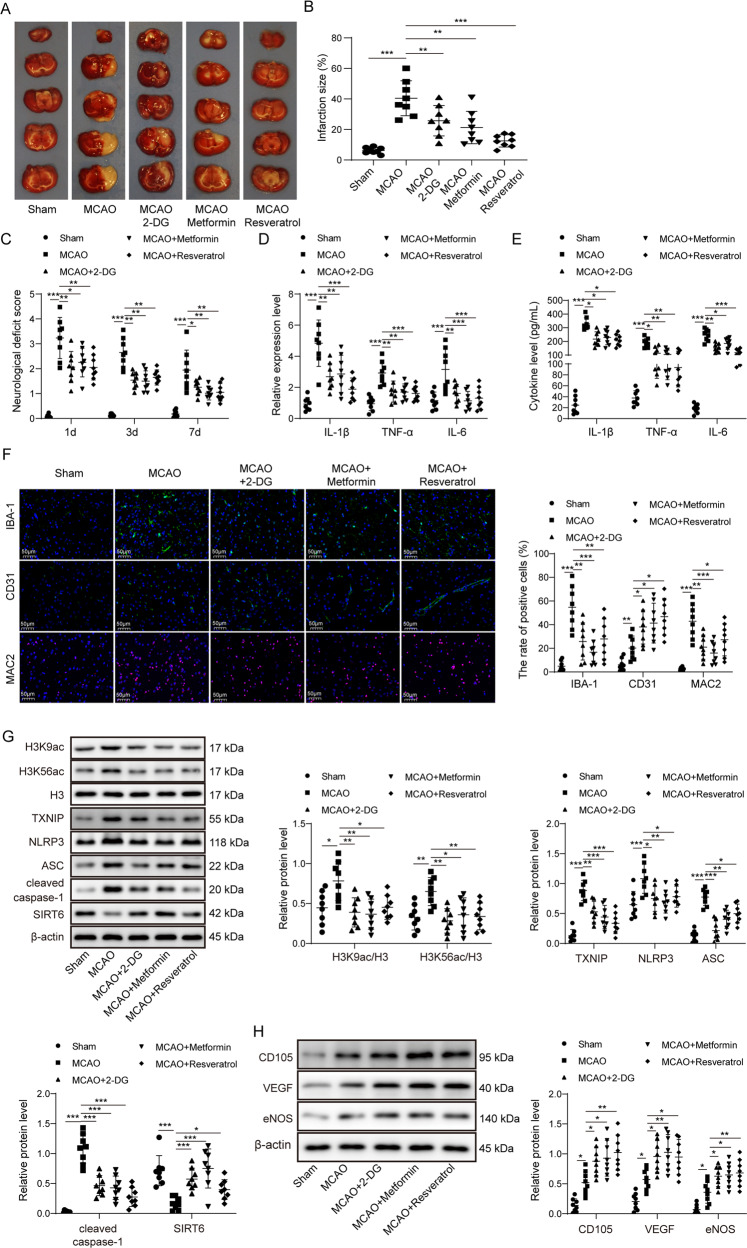
ER mimetics exerted similar effects as IF in vivo. **A** TTC stained sections illustrating normal (red) and infarct area (white). **B** The size of infarct area was expressed as percentage (%) of brain volume. **C** Neurological deficit scores using Longa scoring system. **D**, **E** The mRNA and serum levels of cytokines were determined by qRT-PCR and ELISA assay, respectively. **F** Immunofluorescence staining of IBA-1 (Green), CD31 (Green) and MAC2 (Red) in the peri-infarct cortex. Blue, DAPI. Scale bar = 50 μm. **G**, **H** The protein levels of target proteins were determined by Western blotting. β-actin acted as a loading control for Western blotting. For animal studies, *n* = 8/group. *p < 0.05, **p < 0.01, and ***p < 0.001.

To verify the effects of IF in MCAO rats, rats were treated with three different ER mimetics including 2-DG, metformin or resveratrol at 6 h before MCAO surgery. Significant infarct areas were observed in MCAO rats, whereas pre-treatment of ER mimetics markedly reduced infarct volume (Fig. 2A, B). The neurological scores were evaluated over a 7 days period. On day 1, 3, and 7, pre-treatment of ER mimetics consistently illustrated less impairment compared to MCAO alone group (Fig. 2C). Similarly, significant decrease of both mRNA and secretion levels of IL-1β, TNF-α, and IL-6 were found in MCAO rats pre-treated with these ER mimetics (Fig. 2D, E). The effects of cerebral ischemia on IBA-1 and MAC2 expression were attenuated by three ER mimetics, while the CD31 positive vessels appeared much more abundant (Fig. 2F). Consistently, ER mimetics abolished the effects of MCAO, at least in part, on H3K9ac, H3K56ac, TXNIP, NLRP3, ASC, cleaved caspase-1 and SIRT6 expression (Fig. 2G). In addition, MCAO-increased CD105, VEGF, and eNOS were potentiated by these ER mimetics (Fig. 2H). These data indicate that 2-DG, metformin, or resveratrol exert similar effects as IF in vivo.

### ER mimetics protect against OGD/R-induced microglial activation in HAPI cells, and potentiate OGD/R-induced angiogenesis in BMVECs

**Fig. 3:**
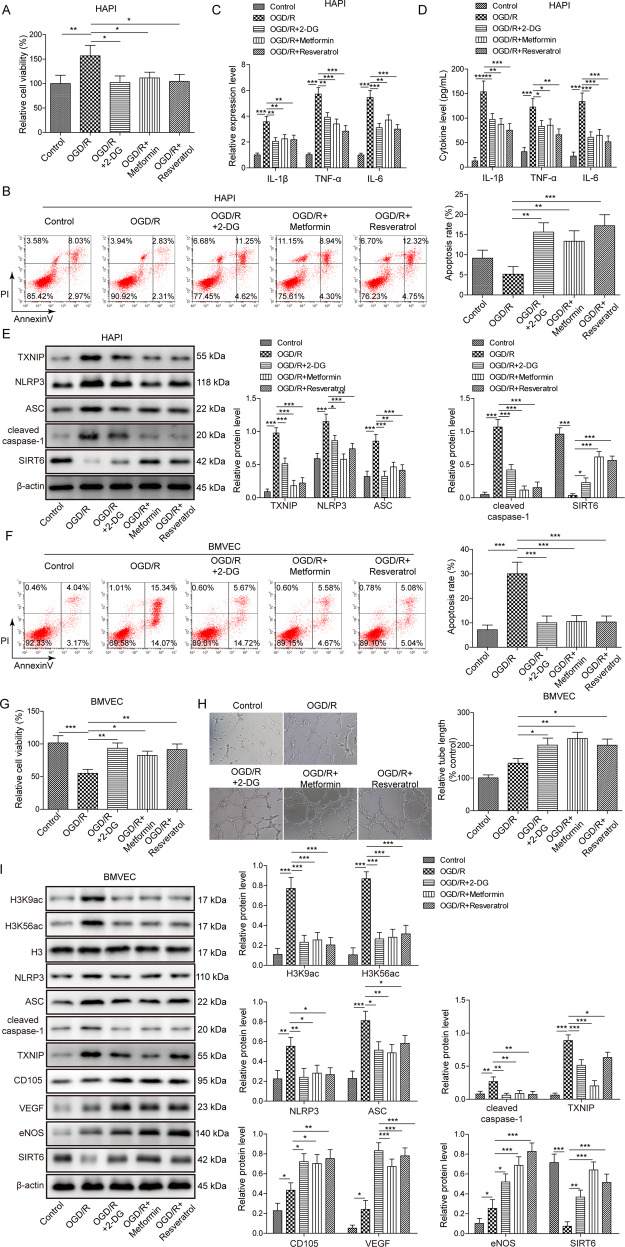
ER mimetics protect against OGD/R-induced microglial activation in HAPI cells, and potentiate OGD/R-induced angiogenesis in BMVECs. HAPI cells or BMVECs were pre-treated with ER mimetics, followed by OGD (2 h)/R (24 h) treatment. **A** The cell viability of HAPI cells was monitored by MTT assays. **B** The cell apoptosis of HAPI cells was monitored by Annexin-V/PI staining. **C**, **D** The mRNA or secretion levels of cytokines in HAPI cells were determined by qRT-PCR and ELISA assay, respectively. **E** The protein levels of target proteins in HAPI cells were determined by Western blotting. **F** The cell apoptosis of BMVECs was monitored by Annexin-V/PI staining. **G** The cell viability of BMVECs was monitored by MTT assays. **H** The angiogenesis of BMVECs was evaluated by tube formation assay. **I** The protein levels of target proteins in BMVECs were determined by Western blotting. β-actin acted as a loading control for Western blotting. *p < 0.05, **p < 0.01, and ***p < 0.001.

We next evaluated the protective effects of ER mimetics in OGD/R cell model which was established to mimic the cerebral I/R in vitro. The cell viability of HAPI cells were increased by OGD/R, whereas 2-DG, metformin and resveratrol abrogated OGD/R-induced cell viability (Fig. 3A). In addition, Annexin-V/PI staining showed that OGD/R-inhibited apoptosis of HAPI cells were reversed by ER mimetics (Fig. 3B). Cytokines were released by activated microglia [18]. Consistent with the in vivo data, OGD/R resulted in a significant induction of IL-1β, TNF-α, and IL-6 on both secretion and mRNA levels, whereas pre-treatment of ER mimetics reduced OGD/R-mediated upregulation of these cytokines in HAPI cells (Fig. 3C, D). Western blotting revealed that OGD/R-induced upregulation of TXNIP, NLRP3, ASC and cleaved caspase-1, as well as downregulation of SIRT6, were partially rescued by ER mimetics (Fig. 3E). Additionally, OGD/R-increased cell apoptotic rate was attenuated by ER mimetics in BMVECs (Fig. 3F). OGD/R reduced cell viability, while BMVECs pre-treated with ER mimetics exhibited a markedly higher cell viability as detected by MTT assays (Fig. 3G). Moreover, BMVECs formed capillary-like structures were abundantly observed in OGD/R group, and pre-treatment of ER mimetics remarkably enhanced tube formation in BMVECs (Fig. 3H). Pre-treatment of ER mimetics potentiated OGD/R-mediated increase of CD105, VEGF and eNOS, and OGD/R-mediated induction of TXNIP, NLRP3, ASC, cleaved caspase-1, H3K9ac, and H3K56ac were attenuated by ER mimetics in BMVECs (Fig. 3I). The expression of SIRT6 was also downregulated by OGD/R in BMVECs, while ER mimetics counteracted the effect of OGD/R on SIRT6 expression (Fig. 3I). Collectively, these data indicate that ER mimetics protects against OGD/R-induced microglial activation in HAPI cells, and potentiates OGD/R-induced angiogenesis in BMVECs. SIRT6 and TXNIP might be involved in the regulation of cerebral ischemia-induced microglial activation and angiogenesis.

### Overexpression of SIRT6 alleviates OGD/R-induced microglial activation in HAPI cells, and potentiates OGD/R-induced angiogenesis in BMVECs

**Fig. 4:**
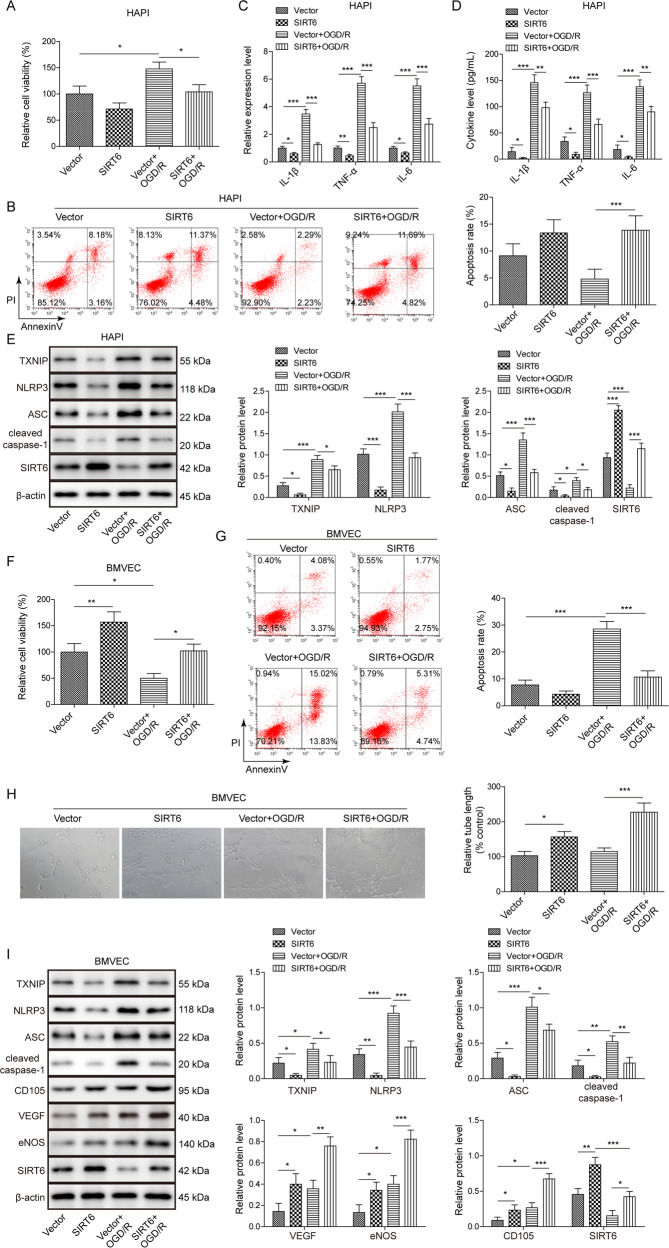
Overexpression of SIRT6 alleviates OGD/R-induced microglial activation in HAPI cells, and potentiates OGD/R-induced angiogenesis in BMVECs. HAPI cells or BMVECs were infected with lentivirus for 48 h prior to OGD/R treatment. **A** The cell viability of HAPI cells was monitored by MTT assays. **B** The cell apoptosis of HAPI cells was monitored by Annexin-V/PI staining. **C**, **D** The mRNA or secretion levels of cytokines in HAPI cells were determined by qRT-PCR and ELISA assay, respectively. **E** The protein levels of target proteins in HAPI cells were determined by Western blotting. **F** The cell viability of BMVECs was monitored by MTT assays. **G** The cell apoptosis of BMVECs was monitored by Annexin-V/PI staining. **H** The angiogenesis of BMVECs was evaluated by tube formation assay. **I** The protein levels of target proteins in BMVECs were determined by Western blotting. β-actin acted as a loading control for Western blotting. *p < 0.05, **p < 0.01, and ***p < 0.001.

To further delineate the role of SIRT6 during cerebral ischemia, SIRT6-overexpressing HAPI cells or BMVECs were subjected to OGD/R treatment. SIRT6 overexpression decreased cell viability and promoted cell apoptosis of HAPI cells under both non-OGD/R and OGD/R conditions, compared with corresponding control (Fig. 4A, B). Overexpression of SIRT6 decreased OGD/R-mediated increase of IL-1β, TNF-α, and IL-6 secretion and expression in HAPI cells. Similar anti-inflammatory effect of SIRT6 was also observed under non-OGD/R condition (Fig. 4C, D). SIRT6 overexpression downregulated TXNIP, NLRP3, ASC and cleaved caspase-1 expression under non-OGD/R condition, and it also reversed the effects of OGD/R on these protein levels in HAPI cells (Fig. 4E). In BMVECs, SIRT6 overexpression promoted cell viability, and the adverse effects of OGD/R on cell viability were ameliorated by SIRT6 (Fig. 4F). SIRT6 overexpression exerted an opposite effect on cell apoptosis in BMVECs (Fig. 4G). Moreover, SIRT6 overexpression promoted tube formation, and OGD/R-induced tube formation was exacerbated by SIRT6 (Fig. 4H). In line with these findings, SIRT6 overexpression decreased TXNIP, NLRP3, ASC and cleaved caspase-1 expression, but increased VEGF, eNOS and CD105 expression under non-OGD/R condition in BMVECs (Fig. 4I). Overexpression of SIRT6 also abolished OGD/R-mediated upregulation of TXNIP, NLRP3, ASC and cleaved caspase-1, and potentiated OGD/R-mediated increase of VEGF, eNOS and CD105 in BMVECs (Fig. 4I). These findings suggest that overexpression of SIRT6 alleviates OGD/R-induced microglial activation in HAPI cells, and potentiates OGD/R-induced angiogenesis in BMVECs. TXNIP might be an important downstream molecule of SIRT6 during cerebral ischemia.

### SIRT6 suppresses TXNIP via deacetylation of H3K9ac and H3K56ac in HAPI cells and BMVECs

**Fig. 5:**
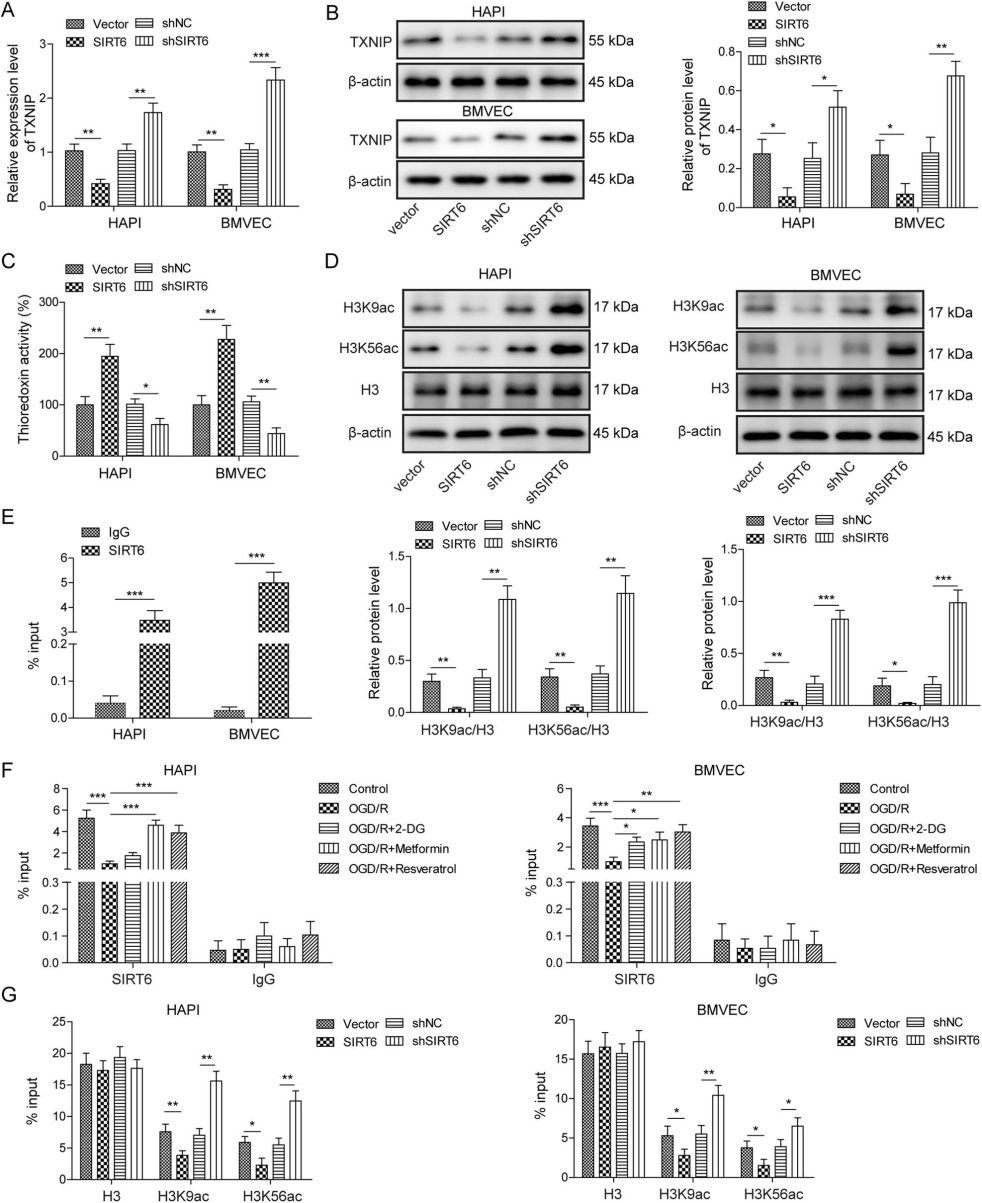
SIRT6 suppresses TXNIP in HAPI cells and BMVECs via deacetylation of H3K9ac and H3K56ac. HAPI cells or BMVECs were infected with lentivirus for 48 h. **A**, **B** The mRNA and protein levels of TXNIP were determined by qRT-PCR and Western blotting, respectively. **C** Thioredoxin reductase activity was measured using commercial kit. **D** The protein levels of target proteins were determined by Western blotting. β-actin acted as a loading control for Western blotting. **E**, **F** The enrichment of SIRT6 at TXNIP promoter was determined by ChIP assay. Normal IgG served as a negative control. **G** The enrichments of total H3, H3K9ac, and H3K56ac at TXNIP promoter were determined by ChIP assay. *p < 0.05, **p < 0.01, and ***p < 0.001.

We next thought to unravel the underlying mechanism by which SIRT6 regulated TXNIP. Gain- and loss of function experiments confirmed that SIRT6 overexpression led to a dramatic reduction of TXNIP, whereas loss of SIRT6 caused a remarkable induction of TXNIP in both HAPI cells and BMVECs (Fig. 5A, B). Additionally, SIRT6 overexpression increased the thioredoxin reductase activity, while SIRT6 silencing inhibited the thioredoxin reductase activity in both HAPI cells and BMVECs (Fig. 5C). It is worth noting that silencing SIRT6 had no effect on SIRT1, SIRT3, HDAC1, HDAC2, and HDAC6 mRNA levels (Supplementary Fig. S1), supporting that shSIRT6 specifically knockdown SIRT6 in both HAPI cells and BMVECs. Additionally, H3K9ac and H3K56ac were significantly decreased in SIRT6-overexpressing HAPI cells and BMVECs compared to control cells (Fig. 5D). In contrast, lack of SIRT6 resulted in an increase on H3K9ac and H3K56ac levels. No significant changes in H3 protein levels were observed in these cells (Fig. 5D). Previous study has illustrated that SIRT6 is highly enriched at the transcriptional start site (TSS) of TXNIP to inhibit TXNIP transcriptionally in beta cells [12]. Similarly, a significant enrichment of SIRT6 was found in the promoter region of TXNIP in both HAPI cells and BMVECs as detected by ChIP assay (Fig. 5E). In addition, OGD/R decreased the enrichment of SIRT6 at TXNIP promoter, whereas ER mimetics partially reversed this effect in both HAPI cells and BMVECs (Fig. 5F). Moreover, we detected a significant decrease of H3K9ac and H3K56ac enrichments at TXNIP promoter in SIRT6-overexpressing HAPI cells and BMVECs. While H3K9ac and H3K56ac ChIP signals were markedly elevated in SIRT6-knockdown HAPI cells and BMVECs (Fig. 5G). No significant change was observed on the enrichment of total H3 at TXNIP promoter in these cells (Fig. 5G). Taken together, these data suggest that SIRT6 suppresses TXNIP expression in HAPI cells and BMVECs via deacetylation of H3K9ac and H3K56ac at TXNIP promoter.

### ER mimetics modulates OGD/R-induced microglial activation and angiogenesis partially via SIRT6

**Fig. 6:**
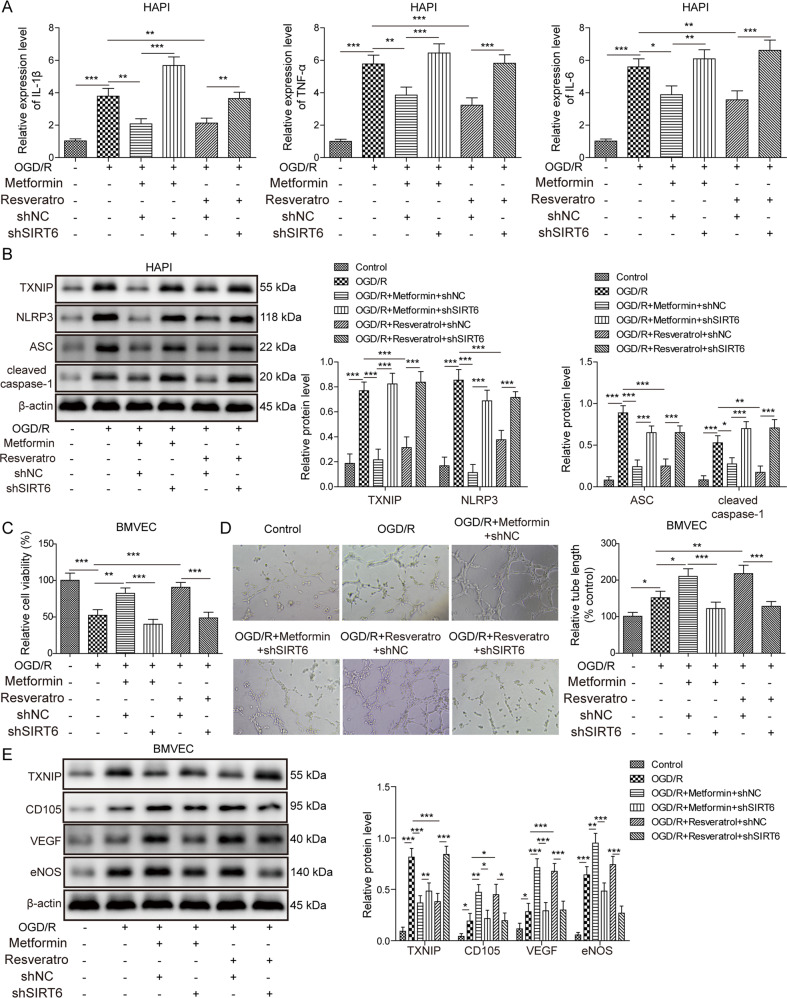
ER mimetics modulates OGD/R-induced microglial activation and angiogenesis partially via SIRT6. shNC or shSIRT6 lentivirus-infected HAPI cells and BMVECs were pre-treated with vehicle control, metformin or resveratrol for 12 h, followed by OGD/R treatment. **A** The mRNA levels of cytokines in HAPI cells were determined by qRT-PCR. **B** The protein levels of target proteins in HAPI cells were determined by Western blotting. **C** The cell viability of BMVECs was monitored by MTT assays. **D** The angiogenesis of BMVECs was evaluated by tube formation assay. **E** The protein levels of target proteins in BMVECs were determined by Western blotting. β-actin acted as a loading control for Western blotting. *p < 0.05, **p < 0.01, and ***p < 0.001.

To gain mechanistic insights into how SIRT6 mediated the effects of ER mimetics during cerebral ischemia, shNC or shSIRT6 lentivirus-infected HAPI cells and BMVECs were pre-treated with vehicle control, metformin (10 μM) or resveratrol (10 nM) for 12 h, followed by OGD/R treatment. ER mimetics metformin or resveratrol successfully reversed the OGD/R-induced upregulation of IL-1β, TNF-α, and IL-6, and the rescue effects were abrogated in SIRT6-knockdown HAPI cells (Fig. 6A). In addition, metformin or resveratrol attenuated OGD/R-induced upregulation of TXNIP and NLRP3 activation, whereas SIRT6 deficiency abolished the effects of metformin or resveratrol on the expression of TXNIP and NLRP3 inflammasome signaling (Fig. 6B). Furthermore, the effects of metformin or resveratrol on cell viability and tube formation were also attenuated in OGD/R-treated SIRT6-knockdown BMVECs (Fig. 6C, D). The protein levels of CD105, VEGF and eNOS were further elevated while TXNIP was dropped upon metformin or resveratrol treatment compared to OGD/R group, while silencing of SIRT6 partially diminished this effect (Fig. 6E). These findings indicate that ER mimetics modulates OGD/R-induced microglial activation and angiogenesis partially via SIRT6.

## Discussion

ER has been recognized as a promising natural approach to overcome age-related diseases including stroke [3, 19]. ER protects against cerebral I/R-induced brain injury by inducing neuroprotective factors and suppressing inflammatory processes [19]. Consistently, our findings showed that the neurological impairments in MCAO IF rats were significantly improved compared with MCAO AL rats. IF promoted angiogenesis and inhibited microglia activation, thereby ameliorating cerebral I/R injury. Emerging evidence suggested that microglia either exacerbated or ameliorated injury following ischemic stroke, depending on the activation signal [20]. In addition, it has been illustrated that microglia was implicated in postnatal retinal angiogenesis [21]. Microglial, especially activated microglial, contributed to angiogenesis and vascular function hemostasis of co-cultured retinal microvascular endothelial cells [22]. On the other hand, it is well-established that cerebral I/R induced a neuroinflammatory response in central nervous system (CNS). In response to cerebral ischemia, microglia are activated, thus resulting in the release of cytotoxic and/or pro-inflammatory mediators [23]. They may lead to decreased cell viability of BMVECs. A number of studies have demonstrated that the cell viability of BMVECs was impaired by OGD/R [24], while tube formation is potentiated in OGD/R group [25]. Mechanistically, Notch and Wnt/β-catenin pathways are activated after cerebral I/R or OGD/R stimulation, thereby facilitating angiogenesis [26]. These findings support that angiogenesis is a self-healing process after cerebral ischemia. In this study, we demonstrated that ER mimetics or SIRT6 overexpression protected BMVECs against HAPI cell activation-induced cytotoxicity. However, the association between microglial activation and angiogenesis during cerebral I/R injury remain elusive. Further study is needed to further unravel the mechanism by which IF or ER mimetics activates microglia during ischemic stroke, and the crosstalk between angiogenesis and microglia activation merits further investigation.

Besides IF regimen, three ER mimetics, namely 2-DG, metformin or resveratrol, were shown to exert similar effects as IF in vivo. It is worth noting that AL rats were much heavier than IF rats. However, the body weights of rats were similar in ER mimetics in vivo model, indicating that weight-dependent effect was negligible. Growing evidence has illustrated the neuropharmacological actions of 2-DG, metformin or resveratrol in stroke. For instance, the glucose analogue 2-DG decreased ischemic brain damage and improves behavioral outcome [27]. The well-known first-line anti-diabetic drug metformin exerted beneficial effects on stroke via AMPK activation [28]. Recently, resveratrol has been shown to protect ischemic injury via JAK/ERK/STAT signaling or gut-brain axis [29, 30]. Unfortunately, the mechanisms by which these ER mimetics passed the blood-brain barrier (BBB) and exerted the neuroprotective effects remain largely elusive. Different molecular mechanisms underlying the effects of these ER mimetics have been demonstrated. For instance, it has been reported that 2-DG elicited cancer cell death via blocking glycolysis, inhibiting N-linked glycosylation or activating autophagy [31, 32]. Additional studies were required to delineate whether these mechanisms can also be applied in HAPI cells or BMVECs. In this study, we have demonstrated that ER mimetics exerted neuroprotective effects via targeting SIRT6, thereby modulating TXNIP and NLRP3 inflammasome activation. ER mimetics exerted the beneficial effects directly in vitro, however, the conditions are much more complicated in vivo. Considering that the in vitro study may not replicate the in vivo condition, future studies are need to corroborate our findings in SIRT6-deficient mice.

Sirtuin family consists of seven highly conserved sirtuins (SIRT1-7) [33]. Most attentions have been focused on SIRT1 given its well-characterized role in aging and ER-mediated beneficial effects. ER attenuated ischemic injury via inducing SIRT1 synthesis [34]. A recent study in APP mutant model of Alzheimer’s disease (AD) has shown that IF reduced hippocampal neuron hyperexcitability and ameliorated cognitive deficits in a SIRT3-dependent manner. Similarly, our data illustrated the critical role of SIRT6 following OGD/R in which the protective effects of ER mimetics were significantly attenuated in SIRT-6-knockdown cells, possibly through regulating TXNIP, NLRP3, and VEGF signaling in vitro. Our data provided additional evidence for the neuroprotective roles of sirtuins in stroke. It is worth noting that the function of SIRT6 in CNS is complicated. A study has demonstrated that SIRT6-induced autophagy also contributed to oxidative stress-induced neuronal damage in human neuroblastoma cell line SH-SY5Y cells [35], indicating that the neuroprotective or neurotoxic role of SIRT6 might depend on cell type. Moreover, SIRT6 has been shown to regulate global protein synthesis via the transcriptional factor Sp1, independent of its deacetylase activity [36], indicating that alternative SIRT6-mediated transcriptional pathway(s) might also be involved in ER-induced neuroprotection. The alternative mechanism and/or the crosstalk among different pathways should be investigated in future research.

TXNIP is originally identified as an inhibitor in thioredoxin (TRX) system and plays an important role in redox homeostasis. TXNIP has been recognized as a crucial link between oxidative stress and inflammation activation in neurons [37]. In response to Reactive oxygen species (ROS), TXNIP rapidly binds to NLRP3 inflammasome, thereby triggering inflammasome activation [37]. This study demonstrated that IF or ER mimetics led to a marked induction of SIRT6, alone with the reduction of TXNIP in vivo, and SIRT6 suppressed TXNIP via deacetylation of H3K9ac and H3K56ac. Additionally, a recent report has demonstrated that depletion of SIRT6 led to an induction of TXNIP in SIRT6 knockout ESCs, however, no significant enrichment of H3K9ac or H3K56ac has been observed on TXNIP locus [38]. This discrepancy suggested that the regulatory mechanism by which SIRT6 regulated TXNIP might be different among ESCs, HAPI cells and BMVECs. Additionally, our findings revealed that SIRT6 inhibited NLRP3 activation in HAPI cells and BMVECs, indicating that IF or ER mimetics exerted the protective effects via SIRT6-mediated TXNIP/NLRP3 axis. Consistently, recent studies demonstrated the protective effects of targeting TXNIP/NLRP3 inflammasome in various disorders [39]. Moreover, angiogenesis is an important therapeutic concept for stroke treatment as generation of new vessels enhance oxygen and nutrient supply to ischemic area, and facilitate neurogenesis [40]. TXNIP is increasingly implicated as a key regulator of angiogenesis [15, 16], however, the role of TXNIP in angiogenesis remains unclear. Most studies showed that enhanced TXNIP downregulated VEGF production [41], while some demonstrated that TXNIP was required for VEGF/VEGFR2 angiogenic signal but it did not affect VEGF levels [42]. Some researchers reported that silencing of TXNIP improved ischemia-induced revascularization in metabolic disorders [43]. Consistent with previous studies, our study showed VEGF and eNOS were negatively correlated with TXNIP, indicating that TXNIP might be involved in angiogenesis via VEGF signaling in BMVECs. These findings indicate that TXNIP is a key player in both post-ischemic inflammation and angiogenesis.

In conclusion, we have demonstrated that ER-mediated upregulation of SIRT6 inhibited microglia activation and potentiated angiogenesis in cerebral ischemia via suppressing TXNIP in vitro.

## Materials and methods

### Animals, diets, and MCAO reperfusion model

All animal study protocols were approved by Xiangya Hospital, Central South University. Male Sprague-Dawley rats (6-week-old, 220–240 g b.w, Hunan SJA laboratory animals, Changsha, China) were house in a temperature-controlled environment under a 12 h light/12 h dark cycle. Rats were randomly divided into the *ad libitum* (AL, *n* = 8) and intermittent fasting (IF, *n* = 8) groups. AL rats were given with a free access to food and water. IF rats were fed 24 h every second day. This feeding schedule was applied for 3 months, and rats were then randomly divided into four groups (*n* = 8 per group): Sham AL; Sham IF; MCAO AL and MCAO IF. For ER mimetics treatment, rats (*n* = 8 per group) were treated with either 2-DG (300 mg/kg), metformin (10 mg/kg) or resveratrol (2 mg/kg) by i.v. at 6 h before MCAO surgery. The MCAO surgery was performed as previously described [44]. Briefly, the left common carotid artery (CCA) of rat was exposed by a midline cervical incision after anesthesia. The external and internal carotid arteries were isolated, and a 3-0 nylon suture coated thread was inserted through internal carotid artery to occlude MCA. Rats were subjected to 2 h occlusion and 22 h reperfusion. For Sham groups, the same surgery was performed without the thread inserted. Rats will be excluded if they lose their appetite completely for 24 h or have poor appetite (less than 50% of the normal amount) for 3 days.

### Infarct volume measurement

The brain was harvested, frozen immediately and cut into 2 mm coronal sections throughout the entire brain as previously described [45]. The sections were stained with 2% TTC solution (Sigma-Aldrich, St Louis, MO, USA) at 37 °C for 30 min. The infarct volume was measured by Image J software (NIH).

### Neurological deficits evaluation

The neurological deficits were evaluated according to the Longa neurological scoring system [46]. 0, no neurological deficit; 1, failure to extend forepaw; 2, circling to one side; 3, failing to one side; 4, failure to walk spontaneously and loss of consciousness; 5, death.

### Western blotting

Protein lysate were prepared in RIPA lysis buffer (Pierce, Rockford, USA). 30 μg of total proteins were separated by SDS-PAGE, and transferred onto PVDF membrane (Pierce). After blocking with 5% non-fat milk, the blots were incubated with primary antibody at 4 °C overnight. The blots were incubated with HRP-conjugated secondary antibody (Invitrogen). The signal was detected using ECL substrate (Pierce). The following antibodies were used in this study: anti-TXNIP (#14715; 1:1000), anti-NLRP3 (#15101; 1:1000), anti-ASC (#13833; 1:1000), anti-cleaved caspase-1 (#4199; 1:1000), anti-eNOS (#32027; 1:1000), anti-H3K9ac (#9649; 1:1000), anti-H3K56ac (#4243; 1:1000), anti-Histone H3 (#4499; 1:2000) and anti-β-actin (#3700; 1:1000) antibodies were from Cell signaling technologies (CST, Beverly, MA, USA). Anti-SIRT6 (ab191385; 1:2000), anti-H3 (ab1791; 1:2000) and anti-VEGF (ab46154; 1:1000) antibodies were from Abcam (Cambridge, UK).

### Immunofluorescence

The rat brain tissues (*n* = 8) were collected and immediately frozen in liquid nitrogen. The frozen sections were fixed with 4% paraformaldehyde (PFA) for 10 min, and permeabilized in 0.1% Triton X-100 for 15 min [47]. The sections were blocked with 10% normal goat serum, and incubated with anti-IBA1 (ab5076; 1:500; Abcam) or anti-CD31 (ab182981; 1:300; Abcam) antibody at 4 °C overnight. Sections were incubated with Alexa Fluor 488 or Alexa Fluor 555-conjugated secondary antibody (Invitrogen). Images were obtained using Olympus confocal laser scanning microscope (Olympus Corp. Japan).

### Enzyme-linked immunosorbent (ELISA) assay

The levels of IL-1β (BMS630), TNF-α (BMS622), and IL-6 (BMS625) in serum or cell culture supernatant were quantified using ELISA kits (Invitrogen) [48]. Briefly, serum was prepared with a clotting for 30 min and a centrifugation at 1000 g for 10 min. Cell culture supernatant was prepared with a centrifugation at 1400 rpm for 1 min. Absorbance at 450 nm was measured using a microplate reader.

### Cell culture and OGD/R

Rat microglial cell line HAPI cells were purchased from American Type Culture Collection (ATCC, Manassas, VA, USA) and grown in DMEM supplemented with 10% FBS (Gibco, Thermo Fisher Scientific). HAPI cells were authenticated by STR profiling, and cells were tested without contamination with mycoplasma. Rat brain microvascular endothelial cells (BMVECs) were isolated and cultured as described [49]. The beaded microvessel fragment and individual endothelial cells were cultured in F12/DMEM containing 20% FBS (Gibco), 3 mg/ml glucose, 0.58 mg/ml L-glutamine, 100 U/ml penicillin and 100 μg/ml streptomycin. For OGD/R treatment, HAPI cells or BMVECs were maintained in serum/glucose-free medium or glucose-free medium supplemented 2% FBS in a humidified incubator with 95% N_2_ and 5% CO_2_ for 2 h, respectively. Thereafter, cells were then returned to normal medium with 5% CO_2_ in air for 24 h. The doses of ER mimetics were determined as previous described [50–52].

### Lentiviral transfection

The full-length of rat SIRT6 were cloned into pLVX vector (Clontech Laboratories, Mountain View, CA, USA). A pair of 59-nt long oligos (5’-ATTCGTGTAAGACGCAGTACGTGTTCAAGAGACACGTACTGCGTCTTACACTTTTTTG-3’ and 5’-AATTCAAAAAAGTGTAAGACGCAGTACGTGTCTCTTGAACACGTACTGCGTCT TACACG-3’), encoding a 19-nt long rat sh-SIRT6 was obtained from GenePharma (Shanghai, China). A scramble shRNA was used as negative control (sh-NC). Lentivirus production was performed in HEK293T cells using Xfect transfection reagent (Clontech Laboratories). HAPI cells or BMVECs were infected with lentivirus for 48 h.

### RNA isolation and quantitative RT-PCR (qRT-PCR)

Cells or tissues were lysed with Trizol reagent (Invitrogen). Total RNAs was subjected to reverse transcription using PrimeScript RT reagent (TaKaRa, Dalian, China) [53]. qRT-PCR was performed using SYBR Premix Ex Taq II (TaKaRa). Amplification conditions were as follows: 95 °C for 10 min followed by 45 cycles consisting of 95 °C for 15 s, 58 °C for 30 s and 68 °C for 60 s. GAPDH was used as an internal control. The mRNA levels of target genes were determined using the 2^–ΔΔCt^ method. The primers used in qRT-PCR were listed in Table 1.

**Table 1:** The primers used in qRT-PCR.

Pirmer	Sequence 5’−3’
IL-β sense	CAGCAGCATCTCGACAAGAG
IL-β anti-sense	CATCATCCCACGAGTCACAG
TNF-α sense	AGTCCGGGCAGGTCTACTTT
TNF-α anti-sense	GGCCACTACTTCAGCGTCTC
IL-6 sense	CCACCCACAACAGACCAGTA
IL-6 anti-sense	AACGGAACTCCAGAAGACCAG
TXNIP sense	AGTTACCCGAGTCAAAGCCG
TXNIP anti-sense	TCTCGTTCTCACCTGTAGGC

### Chromatin immunoprecipitation (ChIP) assay

ChIP assay was performed using Pierce Magnetic ChIP kit (Pierce). In brief, HAPI cells and BMVECs were crosslinked with 1% formaldehyde and harvested. Chromatin fragments were prepared by MNase digestion, and incubated with anti-SIRT6 (ab191385; 3 μg, Abcam), anti-H3 (ab1791; 2 μg, Abcam), anti-H3K9ac (#9649; 10 μl; CST), anti-H3K56ac (ab195478; 5 μg, Abcam) antibody or corresponding normal IgG. DNA was further purified and analyzed by qRT-PCR. Amplification conditions were as follows: 95 ˚C for 1 min, 40 cycles consisting of 95 ˚C for 30 s, 65 ˚C for 30 s and 72 ˚C for 30 s, and 72˚C for 5 min. The following primers were used: Forward 5’-GGCACAACCAGCTGGTTGAA-3’; Reverse 5’-TGAGCCGAGTGGGTTCAAGA-3’.

### Tube formation assay

Briefly, BMVECs were trypsinized and resuspended in culture medium at 4 × 10^5^ cells/ml. Cells were then plated onto Matrigel (BD Biosciences, San Jose, CA, USA)-coated 24-well plate (300 μl/well) and incubated for 24 h. The capillary-like structures were photographed using Olympus inverted microscope (Olympus).

### MTT assay

Cell viability was monitored by MTT assay as described [54]. HAPI cells and BMVECs were plated in 96-well plate prior to the treatment. MTT solution (20 μl/well, Sigma-Aldrich) were incubated with treated cells for 4 h at 37 °C. MTT formazan crystals were resuspended in 150 μl DMSO. Absorbance was measured at 490 nm using a microplate reader (Bio-Rad, Hercules, CA, USA).

### Annexin V-FITC/PI staining

Annexin V-FITC/PI staining was used to monitor cell apoptosis using Cell Apoptosis Kit with Annexin V-FITC and PI (Invitrogen). In brief, cells (1 × 10^5^ cells/ml) were resuspended in binding buffer, followed by the incubation of 5 μl Annexin V-FITC and 5 μl PI. The stained cells were analyzed using flow cytometry (BD Biosciences).

### Thioredoxin reductase assay

Thioredoxin reductase activity was measured using Thioredoxin Reductase Assay Kit (ab83463, Abcam). Briefly, HAPI cells or BMVECs were prepared in cold assay buffer. TNB standards and samples were incubated with Reaction Mix, and absorbance was measured at 412 nm using a microplate reader (Bio-Rad).

### Statistical analysis

Data are presented as the means ± SD in three independent experiments. Statistical significance was determined by using unpaired Student *t* test for two groups or one-way ANOVA when there are more than two groups. All data were analyzed using the SPSS22.0 (SPSS Inc., Chicago, IL, USA). Differences were considered significant if *p < 0.05, **p < 0.01, and ***p < 0.001.

## Supplementary information

Fig. S1

Fig. 5E-ChIP

Fig. 5F-ChIP

Fig. 5G-ChIP

Supplementary Figure legends

Reproducibility Checklist

author contribution form

## Data Availability

All data generated or analyzed during this study are included in this article. The datasets used and/or analyzed during the current study are available from the corresponding author on reasonable request.
