# Supplementary figures and images for: Energy restriction induced SIRT6 inhibits microglia activation and promotes angiogenesis in cerebral ischemia via transcriptional inhibition of TXNIP
Source: Cell Death Dis. 2022 May 11;13(5):449. doi: 10.1038/s41419-022-04866-x (PMC9095711; doi:10.1038/s41419-022-04866-x)

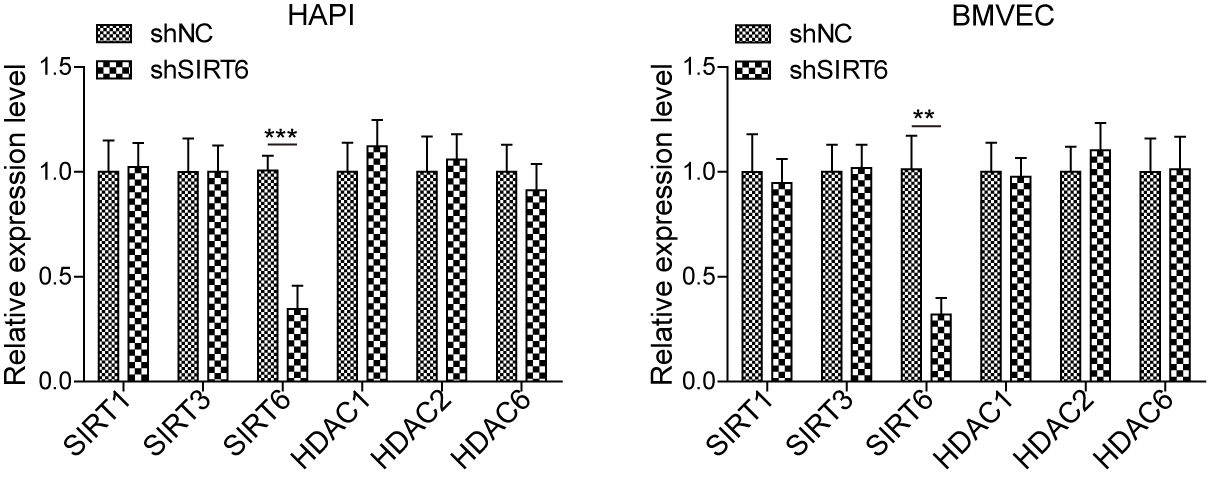

Supplement: Supplementary file 1 — Fig. S1 [file 41419_2022_4866_MOESM1_ESM.tif]

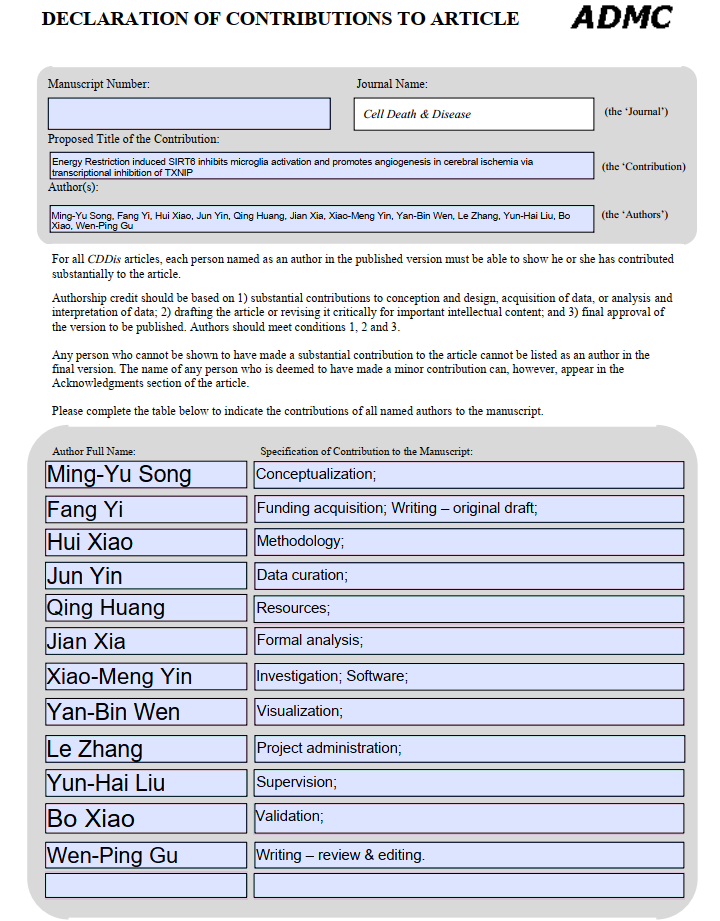


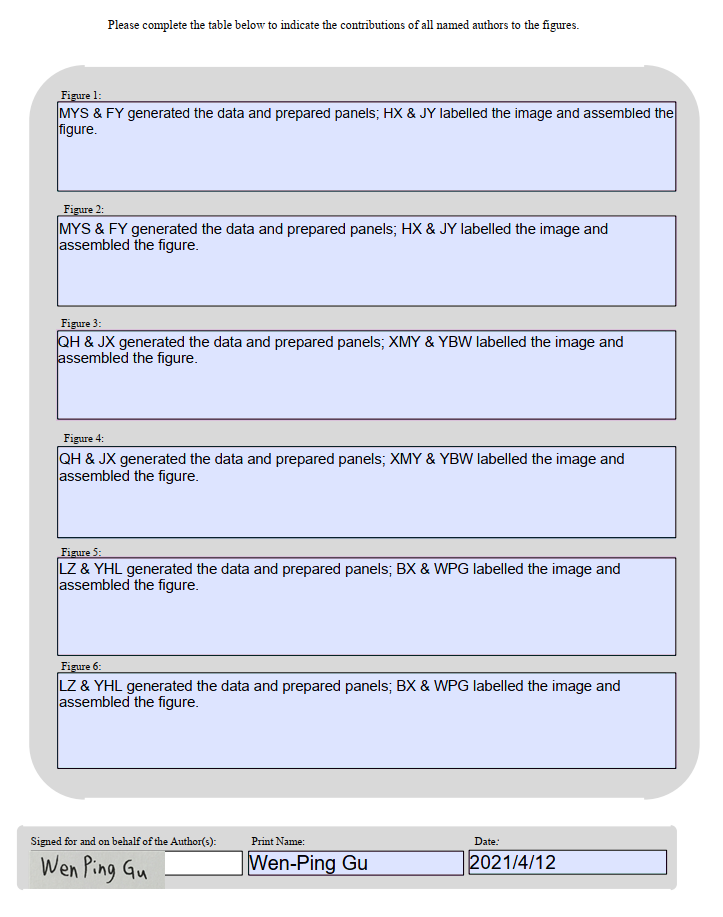

Supplement: Supplementary file 7 — author contribution form [file 41419_2022_4866_MOESM7_ESM.docx]
