# Supplementary material for: Energy restriction induced SIRT6 inhibits microglia activation and promotes angiogenesis in cerebral ischemia via transcriptional inhibition of TXNIP
Source: Cell Death Dis. 2022 May 11;13(5):449. doi: 10.1038/s41419-022-04866-x (PMC9095711; doi:10.1038/s41419-022-04866-x)
Supplement: Supplementary file 5 — Supplementary Figure legends [file 41419_2022_4866_MOESM5_ESM.docx]

**Figure S1. Effects of shSIRT6 on sirtuins and HDACs.** The mRNA levels of SIRT1, SIRT3, SIRT6, HDAC1, HDAC2 and HDAC6 were determined by qRT-PCR. GAPDH served as an internal control.
